# Tensile strength of polyester fiber estimated by molecular-chain extension prior to structure formation

**DOI:** 10.1038/s41598-023-38987-w

**Published:** 2023-07-20

**Authors:** Ren Tomisawa, Mutsuya Nagata, Yumu Otsuka, Toshifumi Ikaga, KyoungHou Kim, Yutaka Ohkoshi, Kazuyuki Okada, Toshiji Kanaya, Hiroo Katsuta

**Affiliations:** 1 Faculty of Textile Science and Technology, Shinshu University, 3-15-1 Tokida, Ueda, Nagano 386-8567 Japan; 2 Institute for Fiber Engineering, Shinshu University, 3-15-1 Tokida, Ueda, Nagano 386-8567 Japan; 3 Material Science Laboratories, Toray Research Center, Inc., 3-3-7 Sonoyama, Otsu, Shiga 520-8567 Japan; 4 Kyoto University, Yoshida Honcho, Sakyo-ku, Kyoto, 606-8501 Japan; 5 Fibers and Textiles Research Laboratories, Toray, 4845 Mishima, Shizuoka, 411-8652 Japan

**Keywords:** Mechanical properties, Process chemistry

## Abstract

The combination of laser irradiation heating and synchrotron X-ray sources has made it possible to observe the fiber-structure development that occurs at sub-millisecond timescales after necking during continuous drawing. Through wide-angle X-ray diffraction (WAXD) and small-angle X-ray scattering (SAXS) analysis of poly(ethylene terephthalate) fibers of three different molecular weights drawn under equivalent stresses, a good correlation was observed between the *d*-spacing of smectic (001ʹ) diffraction extrapolated to the necking point and the strength of the drawn fiber. This indicates that the molecular chains that bear the drawing stress also bear most of the applied stress during tensile testing of the resultant fiber. In addition, considering the drawing-stress dependence of the d-spacing and the molecular weight distribution of the fiber revealed that molecular chains with molecular weights over 23,000 g/mol bear the majority of tensile force applied to the fiber.

## Introduction

Poly(ethylene terephthalate) (PET) fiber accounts for over half of all fibers currently produced. Because of this huge production, numerous studies have been conducted on the structure and properties of PET fibers. Recently, physical-property control has become strongly desired from an environmental viewpoint. Specifically, the tensile strength of the fiber must not only meet use requirements, it should also decrease as quickly as possible after disposal to minimize environmental damage, such as ghost fishing.

Many structural models have been proposed for estimating the physical properties of fibers^1–6^. Most of these assume few-nanometer-sized structures called microfibrils in which crystal and amorphous phases are alternately arranged along the fiber axis. According to these models, tensile force is applied intensively to the taut tie-chains in the amorphous phases connecting crystallites. The properties at small deformations, such as Young’s modulus and thermal shrinkage stress, can be accurately estimated by models using averaged structural parameters, such as crystallinity and molecular orientation. Conversely, the properties at large deformations, in particular fiber strength, are poorly estimated because the initial structure changes with tensile deformation until breakage. However, the amount of taut-tie-chains bearing tensile force is not changed much by tensile deformation. Therefore, we focused on the embryo of the taut-tie-chain, the intermediate so-called ‘smectic phase’^7^. The smectic phase has a fibrillar-shape with an aspect ratio of ~ 10 and is thought to be transformed into microfibrils in the resultant fiber by orientation-induced crystallization^8^. Because the smectic phase comprises bundles of oriented molecular chains, this phase mainly bears the tensile force during the fiber-drawing process prior to orientation-induced crystallization^9^, and these bundles of oriented molecular chains also bear the tensile force applied to the resultant fiber at breakage.

High-molecular-weight polymer is generally used for obtaining high-tensile-strength PET fiber^10^, but excessively high molecular weights negatively affect molecular orientation along the fiber axis because of molecular chain entanglement. This poor molecular orientation decreases the amount of molecular chains bearing the tensile force applied to the fiber, decreasing the tensile strength of the fiber. Accordingly, an appropriate molecular-weight distribution is essential in the design of PET fibers with tailored tensile properties. Particularly, fiber strength is decided by the amount of molecular chains bearing tensile force through the entanglements, and high-molecular-weight chains tend to be those bearing tensile force. Therefore, the concept of a critical chain molecular weight able to bear tensile force would provide a guideline to optimize the molecular-weight distribution of fibers.

The increased availability of synchrotron X-ray sources has made it possible to observe the orientation-induced crystallization behavior of fibers that occurs at sub-millisecond timescales during manufacturing processes such as spinning^11^ and drawing^12^. Furthermore, using the rapid and uniform heating available with CO_2_ laser irradiation on the continuous fiber drawing process, the necking point can be kept stationary. Thus, fiber-structure development can be observed using wide-angle X-ray diffraction (WAXD) and small-angle X-ray scattering (SAXS) patterns taken at different distances from the necking point. The time elapsed after necking can be calculated from the distance divided by the fiber running speed^8^. Moreover, recent improvements in both S/N ratio and diffraction angle resolution have been made possible using an ultra-bright beamline equipped with an undulator and a high-resolution SOPHIAS detector^13–16^, allowing structural changes in the smectic phase to be observed more precisely. Accordingly, good correlation between the amount of smectic phase and the tensile strength of drawn fibers has been observed^14–16^. Furthermore, the apparent modulus was obtained from the change in smectic d-spacing with drawing stress^9^. This indicates that the extension of the molecular chains can be quantitatively estimated from the smectic d-spacing. Therefore, the d-spacing can be used to estimate the fraction of molecular chains that can bear the tensile force applied to the fiber, and it can be used to examine the relationship with the tensile strength more precisely than the amount of smectic phase.

In the present study, we investigated fiber-structure development in PET during laser drawing by simultaneous WAXD/SAXS measurement with a time resolution of 0.1 ms. To investigate the effect of molecular-weight distribution, three kinds of fiber produced by the polymers, H, M, and L (Table 1) having high, moderate, and low average molecular weights, respectively, were analyzed. The smectic d-spacing was obtained from the measurements and used to estimate the amount of molecular chains bearing tensile force. Then, we compared the obtained d-spacing with the mechanical properties of drawn fiber, i.e., strength, elongation, Young's modulus, and thermal shrinkage. In addition, using the drawing-stress dependence of the d-spacing and the molecular-weight distribution of the source polymer, we estimated the critical molecular weight of molecular chains able to bear tensile force.

**Table 1:** Drawing conditions and resultant fiber properties.

Polymer (*M*_*w*_/gmol^−1^)	Drawing stress/MPa	Estimated fiber temperature/°C		Crystallinity by DSC/%	Birefringence	Tensile strength/MPa	Elongation/%	Young’s modulus/GPa	Thermal shrinkage			
									Max. value/%	Temp./°C	Max. stress/MPa	Temp./°C
		0 ms	2 ms									
L (13,800)	92	130	180	46	0.187	680	23	12.4	8	231	55	157
	103	144	202	51	0.194	724	20	12.7	9	238	76	173
M (20,900)	104	151	201	42	0.181	833	25	11.4	13	240	69	176
H (28,300)	63	125	170	37	0.158	774	43	10.0	10	227	40	167
	101	145	192	40	0.181	917	29	11.2	16	243	58	167
	138	157	206	43	0.192	1111	24	12.7	16	242	67	191
	162	165	216	41	0.191	1134	20	12.6	12	245	72	201

## Results and discussion

### Orientation-induced crystallization

**Figure 1:**
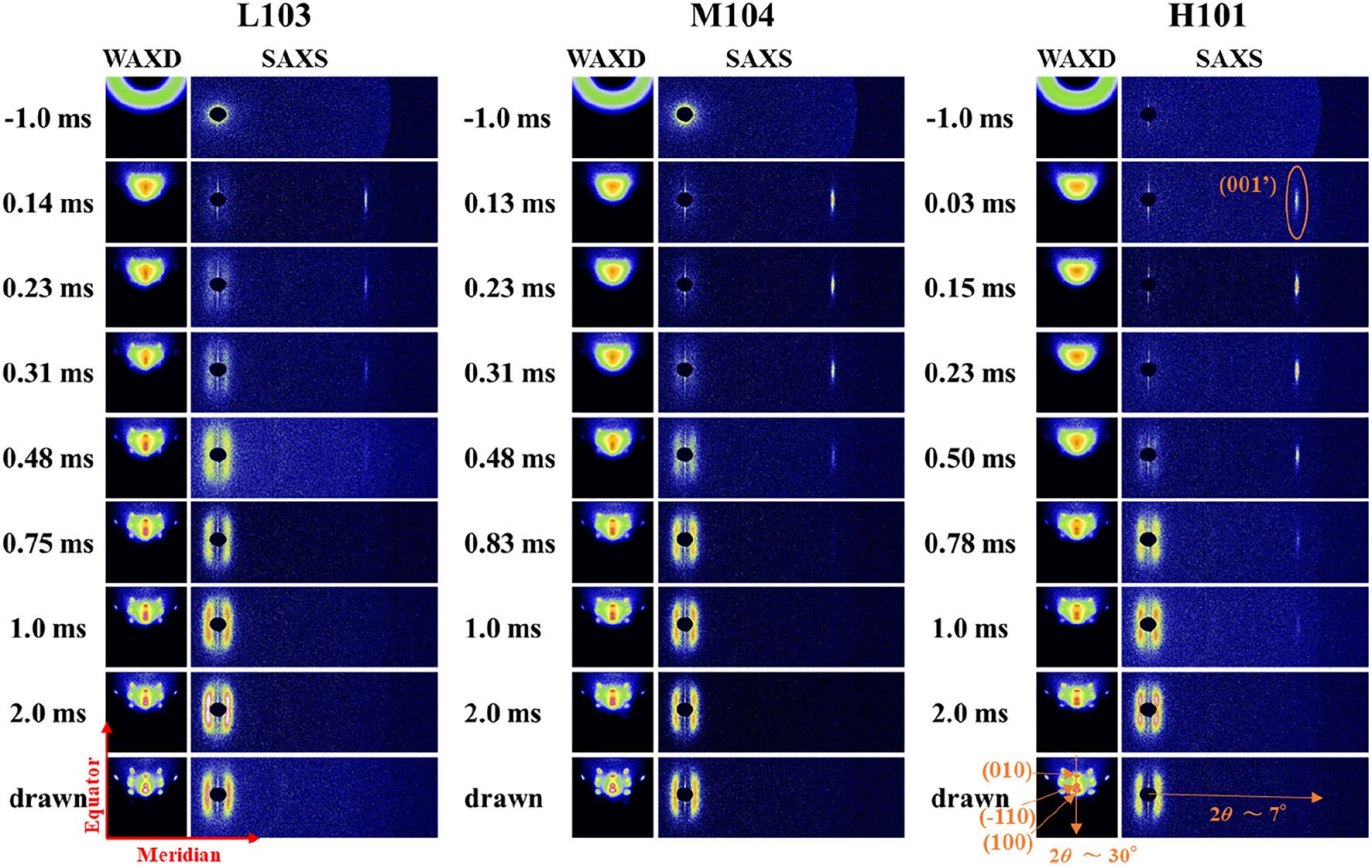
Wide-angle and small-angle X-ray patterns obtained during continuous drawing. The polymer (L, M, H), drawing stress (MPa), and elapsed times after necking are noted in the figure.

WAXD and SAXS patterns obtained at certain elapsed times after necking are shown in Fig. 1. The amorphous halo observed in the WAXD image is concentrated on the equator by the necking, and the (001ʹ) diffraction of the smectic phase^6^ appears on the meridian of the SAXS pattern after necking. Within 1 ms after necking, the (001ʹ) diffraction disappears, while the crystal WAXD diffractions and a four-point SAXS pattern develop. For the H polymer, a stronger smectic (001ʹ) diffraction and a longer elapsed time until dissipation than those for the L polymer are observed (see Fig. E-2a). The appearance of the four-point long-period pattern and crystal diffractions are also delayed for the H polymer. Thus, the orientation-induced crystallization of the H polymer seems to proceed simultaneously with the dissipation of smectic (001ʹ) diffraction. Slower crystallization for high-molecular-weight polymer has already been reported by Kim et al.^17^, but the molecular-weight dependance of the smectic phase was not clarified owing to the insufficient time resolution of the measurement.

**Figure 2:**
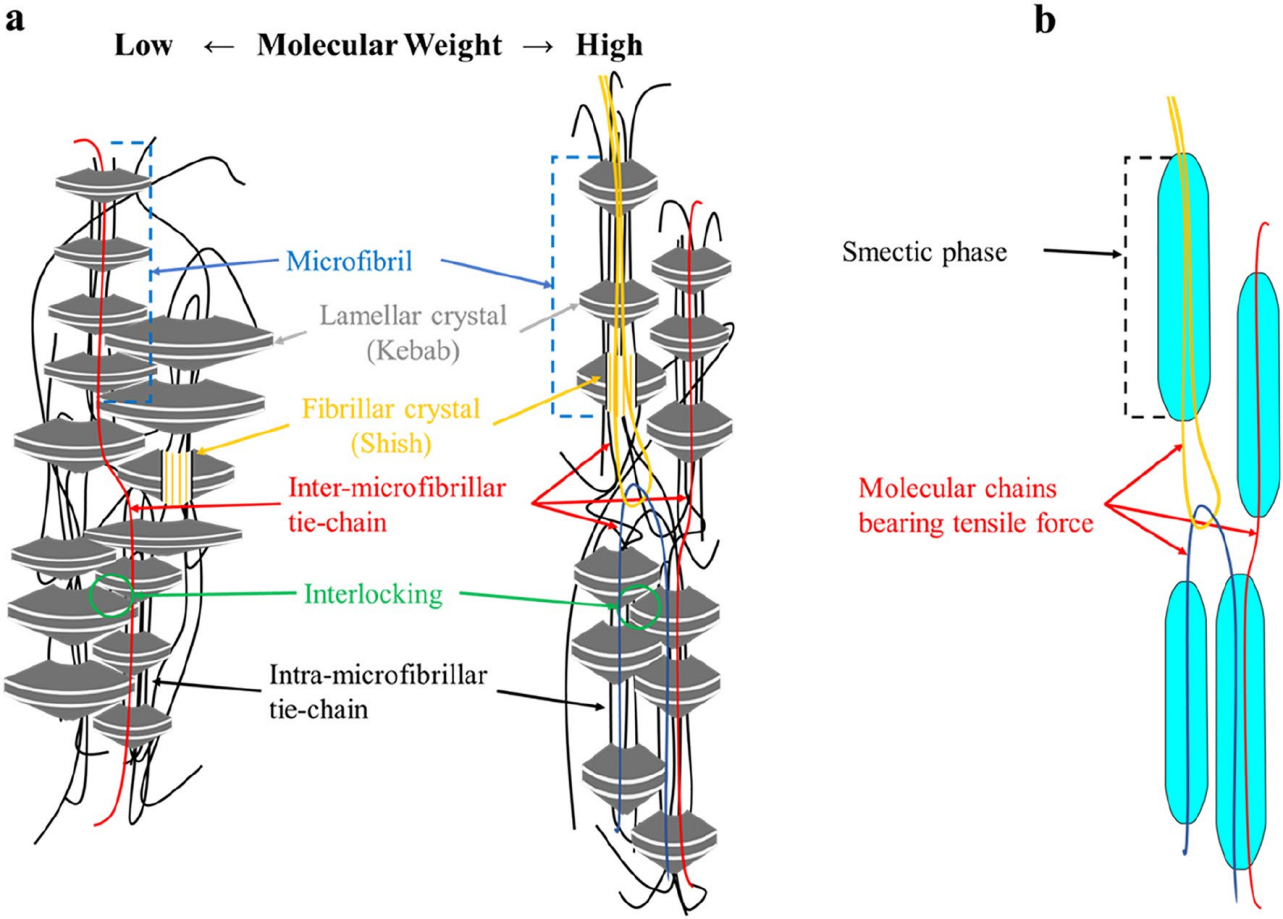
Schematic models for the load-bearing structure; (**a**) microfibrils following Shish–Kebab model^5^ for drawn low- and high-molecular-weight fibers, and (**b**) smectic phase and molecular chains connecting them.

Necking is a plastic deformation brought by the drawing stress, which is borne by the molecular chain network, i.e., molecular chains connected by entanglements. During the plastic deformation, the molecular chains in this network are oriented along the fiber axis, and the strain hardening caused by this molecular orientation terminates the plastic deformation. Because the temperature at the necking point is far higher than the glass transition temperature (Fig. E-1), only the oriented molecular chains can bear external force. Some of the oriented molecular chains forming bundle-like shapes transform into the smectic phase, and other oriented molecular chains connect the smectic phases to each other. In the “shish-kebab” model^5^ shown in Fig. 2a, the smectic phase transforms again into the “shish” parts of microfibrils through crystallization^8^, and the “kebab” parts of the microfibrils are also formed by the crystallization. By this crystallization, the oriented molecular chain bundles are transformed into crystallites and amorphous phases containing numerous intra-microfibrillar tie-chains connecting the crystallites. Therefore, by this crystallization, the molecular chain network bearing the tensile force is transformed into shish molecular chains in the microfibrils and inter-microfibrillar tie-chains connecting microfibrils to each other. Following this model, the delay and suppression of crystallization observed for the H-polymer can be attributed to the higher tension applied to the extended molecular chains in the smectic phase. In contrast, the L-polymer forms less smectic phase despite its higher crystallinity (Table 1). This indicates that most of the L-polymer crystals should be folded-chain lamellar crystals (kebab) comprising molecular chains that bear little tensile force.

### Relationships between the smectic d-spacing and tensile properties

**Figure 3:**
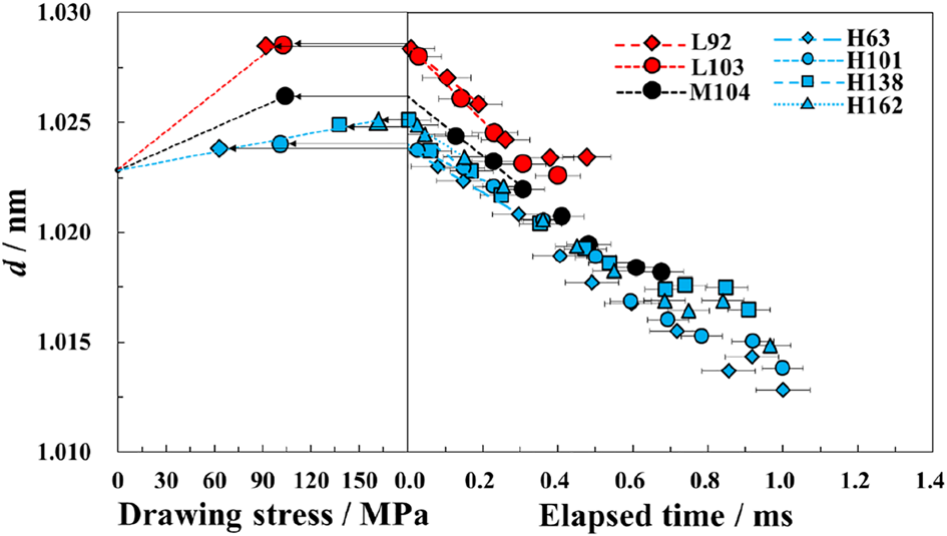
d-spacings (*d*) of smectic (001ʹ) diffraction plotted against elapsed time after necking. The d-spacings are extrapolated to the elapsed time of zero, and the extrapolated values (*d*_0_) are also plotted against the drawing stress. The polymer (L, M, H) and drawing stress (MPa) are noted in the figure.

To investigate the effects of tension applied to the molecular chains in the smectic phase on the resultant fiber properties, we considered the changes in d-spacing (*d*) of the smectic (001ʹ) diffraction obtained from the meridional SAXS profile (Fig. 3). *d* decreases with elapsed time, and at the same elapsed times within 0.3 ms of necking, the fiber of the H-polymer drawn at ~ 100 MPa shows a lower *d* value than the fiber of the L-polymer drawn at an equivalent drawing stress. Because the *d* value represents the chemically repeat-unit length of PET molecule in the smectic phase, the decrease in *d* with elapsed time less than 0.3 ms indicates the relaxation of molecular chains extended by necking, and the lower *d* value of H-polymer drawn at an equivalent drawing stress indicates that more molecular chains in the smectic phase bear tensile force.

**Figure 4:**
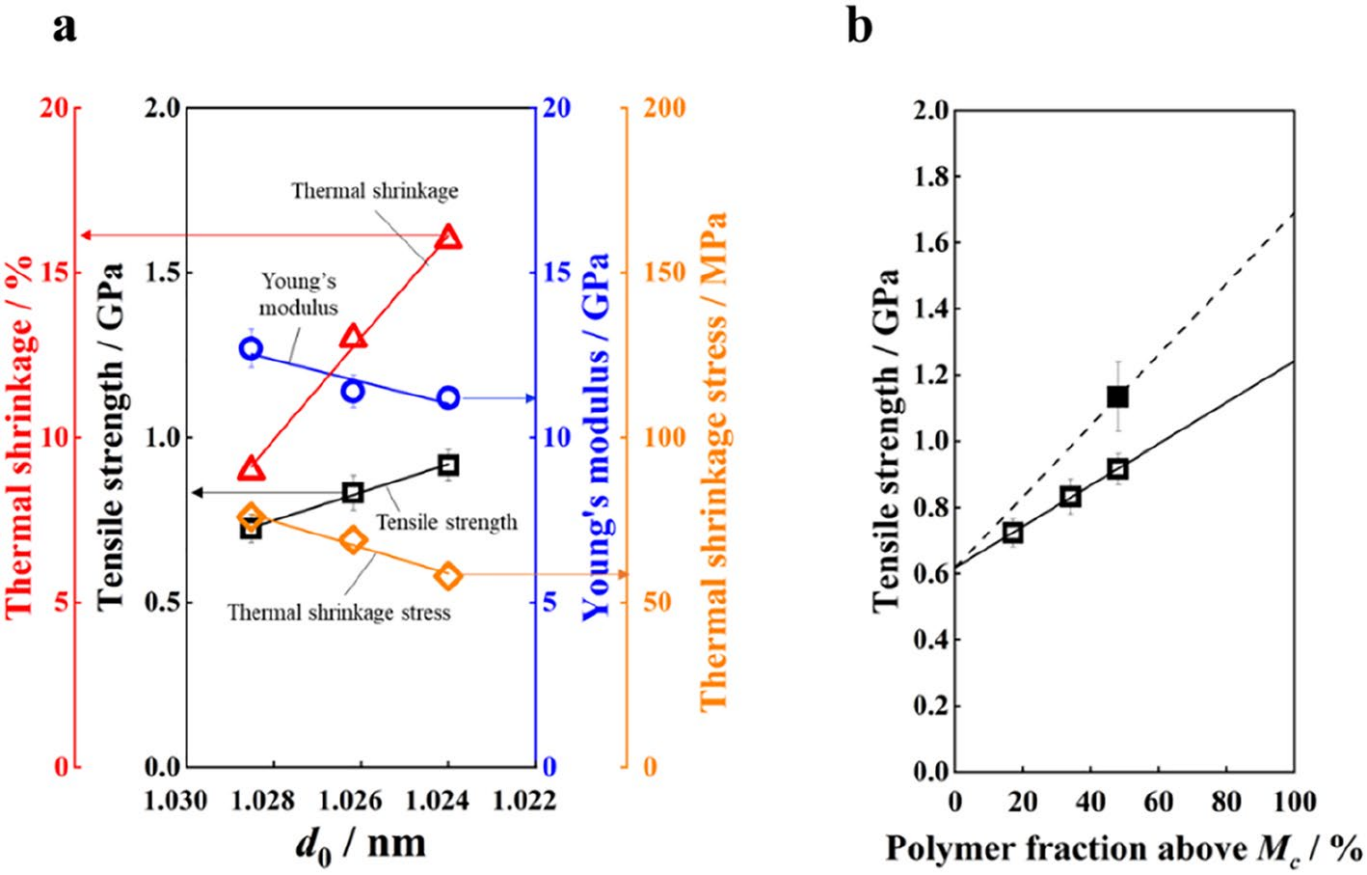
(**a**) Tensile strength (open square), maximum thermal shrinkage (open triangle), maximum thermal shrinkage stress (open diamond), and Young’s modulus (open circle) of drawn fiber plotted against *d*_0_, d-spacing of smectic (001′) diffraction extrapolated to an elapsed time of zero. (**b**) Tensile strength (open square) plotted against the weight fraction of molecules larger than 23,000 g/mol. All properties were measured for fibers obtained at a drawing stress of 100 MPa, except for filled square (162 MPa).

As shown in Table 1, the H-polymer fiber exhibits higher strength, higher thermal shrinkage, lower Young’s modulus, and lower shrinkage stress than the L-polymer fiber, even though their birefringences are very similar. By plotting these properties against *d*_0_, defined as the value of *d* mathematically extrapolated to an elapsed time of zero (Fig. 4a), all the properties show linear relationships. That is to say, the mechanical and thermomechanical properties of drawn PET fiber can be expressed by the molecular chain extension at the neck-drawing point.

The correlation of each physical property with *d*_0_ can be explained as follows (Fig. 2a): The slightly increasing tendency of both Young’s modulus and shrinkage stress properties at small deformations can be attributed to the increased crystallinity of the L-polymer. Interlocking of lamellar crystals (kebab) should suppress the slippage of microfibrils. Conversely, the steep increase in strength and thermal shrinkage of the H-polymer can be attributed to the increase of the shish molecular chains mainly composed of the stress-bearing molecular chains during drawing. This indicates that the molecular chains that bear the tensile force during drawing also bear most of the applied force during tensile testing until fiber breakage. The lesser effect of interlocking on the strength compared with the Young’s modulus and shrinkage stress is due to breakage of the lamellar crystals upon yielding. Furthermore, the lesser effect of interlocking on thermal shrinkage is due to the partial melting of the lamellae crystals at ~ 240 °C, at which point thermal shrinkage becomes maximal.

It is well-known that tensile strength keeps increasing over the draw ratio, even when the birefringence is almost saturated^18^. This indicates that the tensile strength of highly oriented fibers is not just decided by their average molecular orientation and crystallinity, it also depends on the amount and orientation of the molecular chains bearing tensile force. Previously, the amount of these molecular chains has been estimated from the diffraction intensity of the smectic phase^8,16^. The higher diffraction intensity is also observed for the H-polymer in this study (Fig. E-2a). However, the above-mentioned linear relationships between *d*_0_ and the thermomechanical properties allow us to estimate the amount of these chains more quantitatively because the lower *d*_0_ values for the same drawing stress indicate the presence of more molecular chains bearing tensile force.

### Limit strength estimated using the critical molecular weight for bearing tensile force

**Figure 5:**
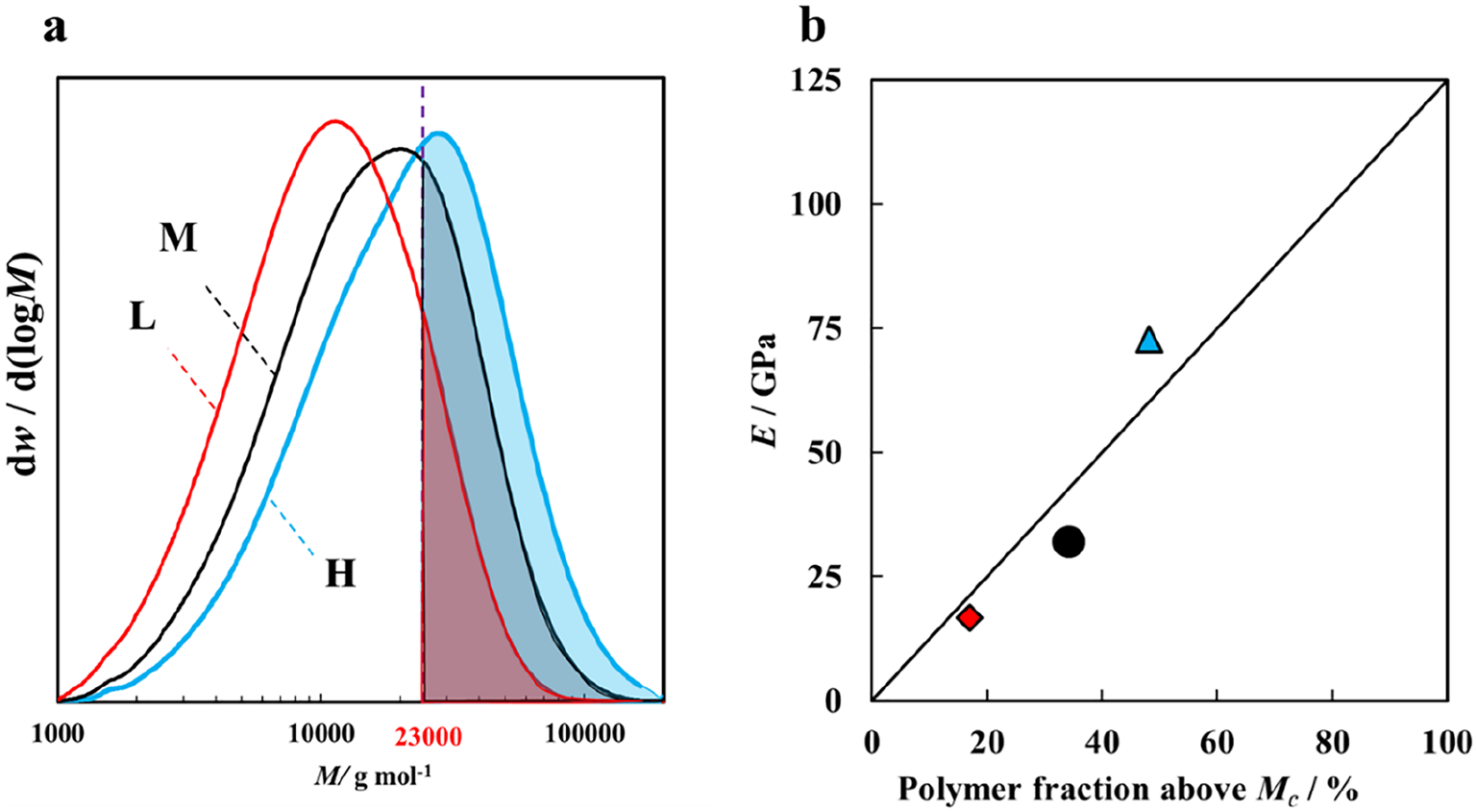
(**a**) Normalized molecular-weight distributions for the three different molecular weight polymers used in this study. (**b**) Apparent elastic modulus *E* obtained for each molecular-weight polymer from Fig. 3 plotted against the weight fraction of molecules larger than 23,000 g/mol. The polymers are L (filled diamond), M (filled circle), and H (filled triangle).

To estimate limiting tensile properties, we concentrated on the relationship between the drawing-stress dependance of *d*_0_ and the molecular weight distribution of the fiber. In a previous study, an apparent modulus of 40 GPa was calculated by plotting *d*_0_ against drawing stress^9^. Following this approach, we calculated the apparent modulus of the H-polymer. In addition, the apparent moduli of the other two polymers were estimated using the intercept obtained for the H-polymer. The apparent moduli should be proportional to the amount of molecular chains bearing tensile force during drawing. Accordingly, the fraction of molecular chains bearing tensile force in a fiber cross-section can be obtained by dividing the elastic modulus by 125 GPa^19^, i.e., the reported theoretical modulus of molecular chains in a crystal. Moreover, higher-molecular-weight chains tend to bear the tensile force in fibers because they form more entanglements. Thus, the fraction of molecular chains bearing tensile force can also be calculated from the molecular-weight distribution (Fig. 5a) as the weight fraction of polymer above a critical molecular weight (*M*_*c*_). The weight fraction of each molecular-weight chain was used for the calculation because the fraction should coincide with the fraction of the chains included in a fiber cross-section. A value of *M*_*c*_ = 23,000 g/mol was determined by a least squares method. Figure 5b shows the relationship between the fractions. The good agreement between the fractions indicates that chains with molecular weight over 23,000 g/mol bear the majority of drawing stress at necking. Moreover, this value of *M*_*c*_ corresponds to a PET chain length of ~ 130 nm, and this is almost twice the maximum length of the smectic phase (50–60 nm; Fig. E-2b). That is to say, a chain length connecting two or more smectic phases may be required to transmit tensile force, as shown by the model in Fig. 2b.

The population of molecular chains over *M*_*c*_ also shows good correlation with the tensile properties of the drawn fiber. Moreover, it can be used for estimating the limiting properties of fibers with different molecular-weight distribution. For example, the attainable maximum tensile strength can be estimated from Fig. 4b. Here, the drawing stress of 100 MPa was the maximum stress for all the polymer fibers drawable, but the fiber of H-polymer can be drawn stably up to 162 MPa. The higher drawable stress of the H-polymer is due to the increased amount of molecular chains bearing tensile force, and it provides a margin for strength increase. Using the strength of a fiber drawn at 162 MPa and the intercept of 620 MPa, an extrapolated strength of 1.7 GPa was obtained by extrapolating to a fraction of 100%. This corresponds to the strength of a fiber in which all the molecular chains bear the drawing stress. This value is somewhat lower than the reported maximum strength of PET fibers of 2.3 GPa^20^ and the extrapolated strength for a surface-defect-free fiber of 1.8–2.1 GPa^14^, presumably because the value was obtained from the strength of fibers without multiple-step drawing nor annealing. However, the strength of 1.7 GPa is only 6% of the theoretical strength^13^. The 620-MPa intercept for strength indicates that more molecular chains bear the external force in tensile tests than in drawing, which may be caused by the testing temperature being far lower than the glass transition temperature, while the drawing temperature is higher. In addition, some loosened inter-microfibrillar tie-chains can be tightened by tensile strain applied during tensile testing. Nevertheless, the almost-three-fold increase in tensile strength with the fraction of molecular chains larger than *M*_*c*_ demonstrates the crucial contribution of the molecular chains to the fiber strength. Thus, if the inter-microfibrillar tie-chains, mainly consisting of molecular chains larger than *M*_*c*_, can be disrupted after the disposal of the fiber, the dramatic decrease in fiber strength would prevent environmental damage such as ghost fishing.

## Methods

### Samples

Fibers used for drawing were prepared by the melt-spinning of three kinds of PET polymers (L, M, and H) supplied by Toray Industries, Inc. The polymers were melt-spun with extruding from a one-hole nozzle at a mass flow rate of 5.0 g/min and taken up at 300 m/min. The spinning temperature was 280 °C for L and M and 310 °C for H. The nozzle diameter and the length/diameter ratio were 1.0 mm and 3, respectively.

The drawing conditions are given in Table 1. The fiber temperature profiles were estimated referring to a previous study^21^, and the absorption coefficient at laser beam wavelengths of 1.149 × 10^4^ m^−1^ for PET were used to estimate the laser irradiation energy. The heat transfer coefficient was estimated using the experimental formula proposed by Kase and Matsuo^22^. The heat of crystallization was calculated from the heat of fusion of drawn fibers measured by DSC. The obtained temperature profiles are summarized in Fig. E-1.

The molecular weight distributions for as-spun fibers were measured by a Waters-Alliance-e2695 equipped with a ShodexHFIP806M column and a Waters-2414 differential refractometer detector. Measurement was conducted at 30 °C using hexafluoroisopropanol supplemented with 0.005 N sodium trifluoroacetate as the eluent at a concentration of 0.83 w/v% and a flow rate of 1.0 mL/min. Narrow-molecular-weight-distribution polymethylmethacrylate was used as a standard. The obtained weight average molecular weights (*M*_*w*_) of the L, M, and H polymer fibers were 13,800, 20,900, and 28,300, respectively. Their molecular weight distributions are shown in Fig. 5a.

### On-line WAXD/SAXS measurement

Fibers were drawn continuously owing to the speed difference between the feed and take-up rollers under heating by CO_2_ laser irradiation^8^. The fiber running speed after necking was fixed at 110 m/min, and the draw ratio was controlled by changing the fiber feed speed. A random polarized laser beam (wavelength, 10.6 µm; spot diameter, 6 mm) was generated by a PIN-30R laser (Onizuka Glass Co., Ltd.) and used to irradiate the running fiber from three different directions. The laser power for each draw ratio was determined to minimize fluctuations in the neck-drawing point. The drawing stress was calculated from the diameter of the drawn fiber and drawing tension, which was measured using a tension meter (HS-1500S, Eiko Sokki Co., Ltd.) with a 1-N pickup installed between the neck-drawing point and take-up roller.

X-ray diffraction patterns of the running fibers were acquired at certain elapsed times after necking. The elapsed time after necking, determined by the distance between the X-ray irradiation point and necking point, was changed by moving the laser irradiation position. For elapsed times of less than 1.0 ms, the distance was measured accurately by video images acquired from the direction coaxial to the X-ray beam using a video camera (WAT-232S type, Watec Co., Ltd.) equipped with a telecentric lens (TV-2S, OPTART Co., Ltd.) with × 2  magnification. In addition to the average distance, the necking point fluctuation width was determined using every tenth still image taken from the video images. A time resolution of 0.11–0.14 ms was calculated from the fiber running speed and the position resolution, which was obtained from the necking point length (0.16–0.29 mm), X-ray beam width (0.04 mm), and the aforementioned necking point fluctuation width (0.01–0.06 mm). This measurement method is described in more detail in a previous report^13^.

The ultrahigh-intensity synchrotron X-ray beamline of SPring-8 BL03XU (FSBL) equipped with an undulator was used in this study. The wavelength of the X-ray beam was 0.10 nm and the beam dimensions were 40 µm × 40 µm along the vertical and horizontal directions. For WAXD and SAXS measurements, the camera lengths were 96 and 393 mm, respectively, the exposure time for both measurements was 0.4 s, and the detector was a 1032 px × 1032 px flat panel detector (50 mm/px) and a direct-detection X-ray SOI-CMOS 2D detector (SOPHIAS; 1.9 Mpx, 30-µm square pixels, imaging area of 26.7 mm × 64.8 mm), respectively^23,24^. This system had integrating-type pixels and was operated at 20 frames/s. To verify the measurement precision, every ten images were acquired, analyzed individually, and then the average and standard deviation were calculated.

### Analysis of X-ray images

The intensity profile along the equatorial direction obtained from each WAXD pattern shown in Fig. 1 was fitted by the three crystal diffraction peaks and a broad amorphous halo, which were all assumed to have Gaussian profiles (Eq. 1).

1 $$I\left(\theta \right)={I}_{0}\;{\text{exp}}\left\{-4\;{\text{ln}}\;2\times {\left(\frac{2\theta -2{\theta }_{0}}{\beta }\right)}^{2}\right\},$$

where 2*θ*_0_, *I*_0_, and *β* are the position, intensity, and full width at half-maximum (FWHM) of each peak and halo, respectively. The equatorial intensity profiles were fitted well by the diffraction and halo profiles. The crystallinity index (*X*_*c*_) was determined as the integrated intensity ratio of the crystal diffraction to the total. The obtained *X*_*c*_ shown in Fig. E-3 started to increase less than 1.0 ms after necking, and it was almost saturated 3.0 ms after necking. Higher *X*_*c*_ values than those at 3.0 ms for the drawn fibers can be regarded as secondary crystallization that proceeds after the termination of primary crystallization. Accordingly, the primary crystallization rate (*K*_*c*_) and the induction time of primary crystallization (*t*_*0*_) were estimated by applying Eq. (2) assuming the *X*_*c∞*_ of *X*_*c*_ at 3.0 ms.

2 $${X}_{c\left(t\right)}=\left\{1-{K}_{c}\;{\text{exp}}\left(t-{t}_{0}\right)\right\}{X}_{c\infty },$$

As shown in Fig. 1, the streak-like smectic (001ʹ) diffraction peak appeared 0.1–0.2 ms after necking, and it faded with the appearance of crystal diffractions. The amount, d-spacing (*d*), and persistence length (*L*) of the smectic phase were evaluated from the integrated intensity along the meridian (*I*), peak position, and widths of the diffraction peak^9^. The integrated intensity (*I*) was normalized by the X-ray irradiation volume. The peak position and width along the meridian were obtained by fitting of the Gaussian function (Eq. 1). The *d*, and *L* values were calculated using the Bragg and Scherrer equations (Eqs. 3, 4, respectively):

3 $$d= \frac{\lambda }{2{\text{sin}}{\theta }_{a}},$$

4 $$L= \frac{K\lambda }{{\beta }_{a}\;\mathrm{cos}\;{\theta }_{a}},$$

where *I*_0_ is the peak intensity, $\lambda$ is the X-ray wavelength, and constant *K* is 1. The half-diffraction angle ($\theta$_*a*_) and integral widths along to the meridian ($\beta$_*a*_) were used for the calculation.

### Birefringence

Birefringence for each fiber was measured using a polarized microscope (BX51-33POC, Olympus Co., Ltd.) with a 546-nm monochromic filter. Tricresyl phosphate was used as the immersion oil. The average and standard deviation of the birefringence were calculated for every ten samples.

### Thermomechanical tests

The tensile properties of the drawn fibers were analyzed by tensile tests using an Autograph AGS-X instrument (Shimadzu Co., Ltd.) equipped with a 50-N load cell. The sample length and elongation rate were 40 mm and 100%/min, respectively. The average and standard deviation of the strength, elongation, and Young’s modulus were calculated for every ten samples. The natural draw ratio (NDR) was defined as the draw ratio at which the tensile stress began to increase again with dissipation of the necking point.

Thermal and mechanical properties were also analyzed by TMA and DSC. TMA was conducted using a thermomechanical analyzer (TMA/SS6100, SII Nanotechnology Inc.) to measure the thermal shrinkage factor and shrinkage stress at heating rates of 5 and 10 K/min, respectively. The sample length was 10 mm for both measurements. DSC was performed using a calorimeter (ThermoPlus2, Rigaku Co., Ltd.) with a heating rate of 10 K/min. Short-cut fibers were used as samples for the measurements. The melting point and crystallinity were determined from the peak position and the heat of fusion in the DSC curve. The heat of fusion of a PET crystal (135 J/g)^25^ was used for calculations.

## Supplementary Information

Supplementary Figures.

## Data Availability

The datasets used and/or analyzed during the current study are available from the corresponding author on reasonable request.
