# Supplementary material for: Tensile strength of polyester fiber estimated by molecular-chain extension prior to structure formation
Source: Sci Rep. 2023 Jul 20;13:11759. doi: 10.1038/s41598-023-38987-w (PMC10359322; doi:10.1038/s41598-023-38987-w)
Supplement: Supplementary file 1 — Supplementary Figures. [file 41598_2023_38987_MOESM1_ESM.pdf]

Supplementary Information

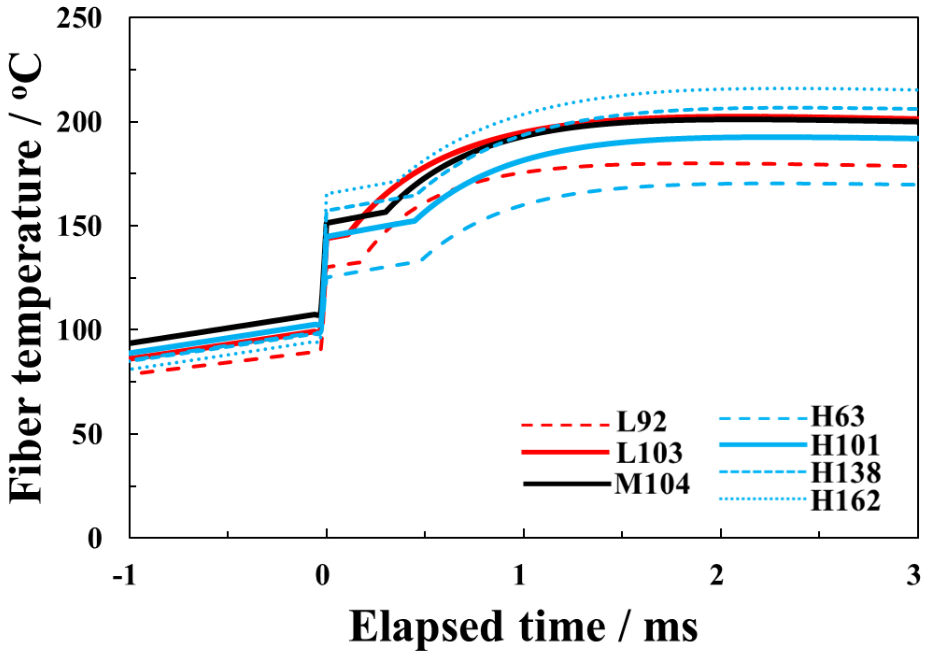

Fig. E-1 All estimated fiber temperature profiles plotted against elapsed time after necking. The polymer (L, M, H) and drawing stress (MPa) are as noted in the figure.

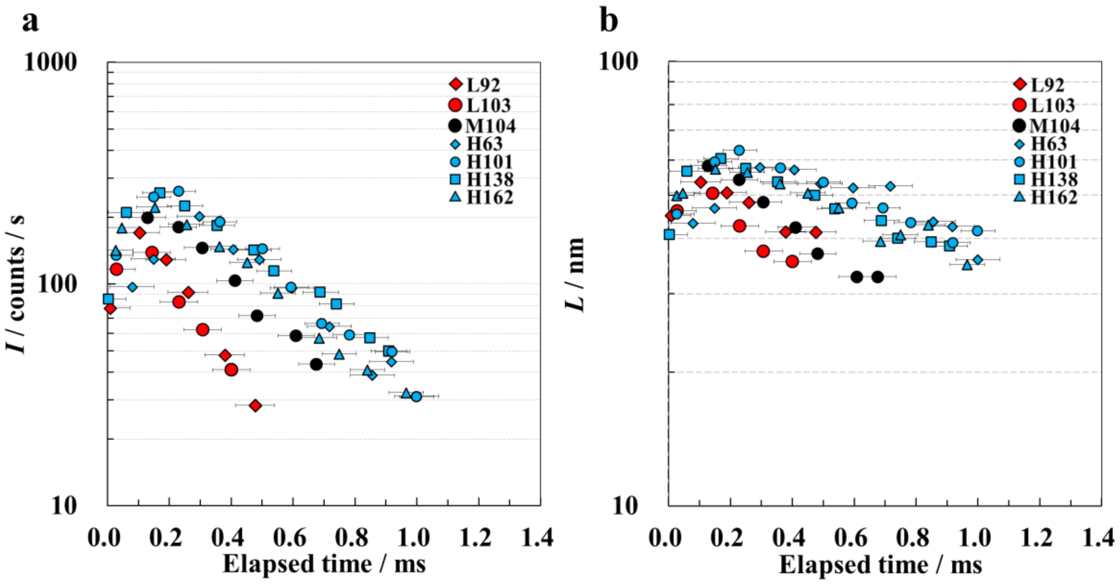

Fig. E-2 **a**, Integrated intensity (*I*), and **b,** persistence length (*L*) obtained from the meridional intensity profile of smectic (001') diffraction plotted against elapsed time after necking. The polymer (L, M, H) and drawing stress (MPa) are as noted in the figure.

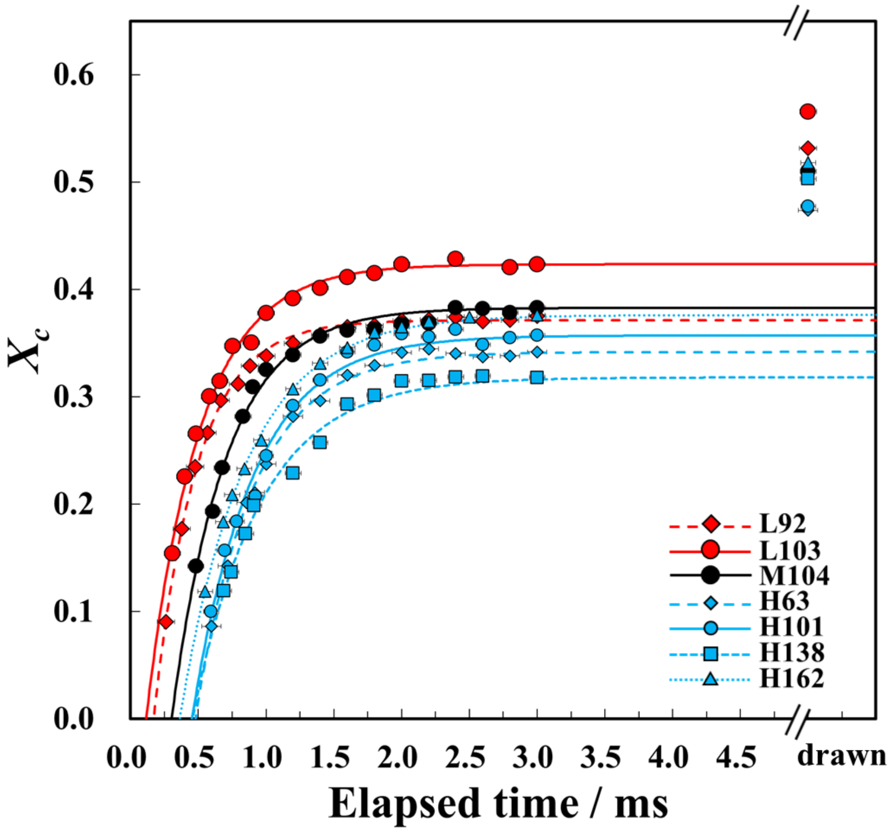

Fig. E-3 Crystallinity indices (X_c) obtained by the integration of equatorial intensity profile plotted against elapsed time after necking. The fitting curves from eq. 2 are also shown. The polymer (L, M, H) and drawing stress (MPa) are as noted in the figure.
